# Cases Read before the Chicago Medical Society

**Published:** 1864-11

**Authors:** Hiram Wanzer

**Affiliations:** Chicago, Ill.


ARTICLE XXXIX.

### CASES READ BEFORE THE CHICAGO MEDICAL SOCIETY. Friday Evening, October 21, 1864.

By HIRAM WANZER, M.D., of Chicago, Ill.

Mr. President and Gentlemen:—I present you three cases this evening; one a premature birth, and two cases of injuries of the head, which I hope will prove interesting to the Society.

CASE I. Mrs. S., aged 22, some time in April last, received a fall upon a chest, injuring the small of the back, near the junction of the dorsal with the lumbar vertebra. She was a primipara—three and a half months advanced in pregnancy. Uterine contractions were induced consequent upon the injury; slight hemorrhage, &c. Large and frequent doses of opium and sugar of lead were given; rest and strict recumbency enjoined which resulted happily in arresting further parturient action. Three months after, I was again summoned to her in labor. Her pains for hours were severe. The undeveloped cervix and rigid os were long dilating. The labor throughout seemed unnatural; when at last she gave birth to a male child, at about six and a half months, with the following pathological appearances:—

At the junction of the dorsal with the lumbar vertebra had been probably a contusion, which resulted in a deep granulating ulcer some three inches in circumference, surrounding the steep edges of this ulceration was an old cicatrix, indicating the reparative process had began; the granulations looked healthy also; and had she gone on to full utero gestation, in my opinion, the process of reparation would have been complete. The point of pathological inquiry here is, as it has been questioned in the Society, do exterior causes acting upon the mother sometimes produce identical pathological results upon her offspring in utero? I answer, I believe they not unfrequently do.

Cases are vivid in my mind, I could recount from recollection, where they have. In this case the mother received a direct blow upon her spine, transmitted to the spine of her infant; this I believe was the case. The abnormal condition of the child might have been diagnosed spina bifida, but there were none of the symptoms present, no faulty formation of the vertebra, no protruding sac. The child died on the second day. The question was ably discussed in the Society by Drs. HOLMES, BARTLETT, and GROESBECK, without arriving at any definite conclusion.

CASE II. J. E., a boy aged five years, was struck by a rail car; it was a gliding blow over the lower jaw, base of the skull, and temporal region without fracture. There was a discharge of blood from the ear, and dilatation of the pupil. He had a convulsion at my first visit, and during the paroxysm there was an alternate protruding and receding of one eye, which phenomenon I could not understand. He continued comatose several days from receipt of injury, showing marked symptoms of cerebral extravasation. He was quite unconscious of his surroundings. The organs of special sensation seemed dormant. The power of deglutition was impaired; when at last he slowly reacted from his coma, and began to take small quantities of concentrated fluid nourishment; his recovery was complete. Dr. Orrin Smith saw the case with me in consultation. This case was treated nearly throughout without medication. It has been my lot the past four years to meet many cases of surgical injuries about the head, and *occasionally* where I have since thought medication did my patient much harm. In traumatic injuries about the head, except severe fracture, and like surgical casualities, I had rather leave the patient to the conservative power of nature than to attempt the administration of much medicine. The point of inquiry has been occasionally with me how to more definitely diagnose between concussion of the brain and cerebral extravasation. One aged physician, emeritus professor of obstetrics in one of our colleges, who has seen much practice, tells me he has seen the same symptoms of coma, as here described, continue two weeks in concussion of the brain without cerebral extravasation; I asked him if his patient died? he said no; that he had founded his conclusions from the fact the pupil of the eye was contracted, and the patient answered coherently.

Case III. Called to James Duffy, aged 24, October 22, 1864, at 11 A.M.; found him lying comatose and hemiplegic. At the junction of the left parietal with the temporal bone there was a circular opening, through soft parts and cranium large enough to admit the finger to the surface of the brain, detecting comminuted fracture of the skull. The bony fragments are before you. Fourteen hours after the injury, assisted by Prof. E. Andrews, the operation for trephining was performed; all the spiculæ of bone were carefully removed. The membranes of the brain were penetrated by them, and also the cerebral substance; one of the branches of the middle meningeal artery was torn across. After cleansing the wound the parts were brought in apposition by interrupted sutures, compress, and bandage. He soon rallied from his coma after the operation, and recognized all around him. After reacting well from the coma and surgical interference, he was removed to Mercy Hospital, where every attention was given him. He died on the fifth day. Drs. BLAKE and BARTLETT performed the *post mortem*, and it was my privilege to be present to record the following notes: There was *post mortem* lividity of the depending portions of the body, also a peculiar goose skin appearance over the surface of the chest. Except the wound, there were no external lesions, ecchymoses, or abrasions found upon the external surface of the head. There were no lesions upon the body or extermities. Upon reflecting the scalp, we found the whole lateral surface of the head, temporal muscle, temporal fascia, and pericranium in an ecchymosed condition, showing he must have received several most violent blows upon the side of the head; upon removing the calvarium we found, as remarked, a terminal branch of the middle meningeal artery wounded. In the interior of the skull surrounding the seat of fracture was blood stain, and a corresponding ecchymosis upon the surface of the dura mater. In the arachnoid sac over the greater part of the left hemisphere there was a thin layer of effused blood, thickest nearest the injury. The parietal layer of the arachnoid showed a high state of inflammatory congestion and engorgement. The veins over the surface of the brain, and the sinuses were all unduly filled with dark blood. All the vessels of the left hemisphere were abnormally injected, together with the vena galeni, choroid plexus, and veins on the left corpus striatum; some effusion of altered lymph or pus was found under the arachnoid on the right side. There was extensive inflammatory softening of the brain from the seat of injury to the left ventricle, so that from the fracture to the ventricle there was a cavity with walls of soft ash-colored pulp. This softening involved the cerebral substance over an area of about two inches. The brain substance between this cavity and the falx cerebri, for an inch downward was the seat of numerous points of extravasation. A thin clot rested in a slit half an inch long in the gray matter; more deeply there was the appearance of capillary apoplexy. The ventricle was filled with bloody serum; the cerebellum had participated also in the inflammatory engorgement.

